# The relationship between personality traits and individual factors with perinatal depressive symptoms: a cross-sectional study

**DOI:** 10.1186/s12884-023-05701-7

**Published:** 2023-05-25

**Authors:** Riccardo Serra, Nicoletta Giacchetti, Francesco Saverio Bersani, Gaia Cappannini, Melania Martucci, Matteo Panfili, Carla Sogos, Franca Aceti

**Affiliations:** Department of Human Neurosciences, Sapienza University of Rome, Viale delle Università, 30, Rome, Italy

**Keywords:** Perinatal period, Woman’s health, Pregnancy, Depression, Family

## Abstract

**Background:**

Pregnancy is a crucial transition moment exposing women to potential mental health problems, especially depressive disturbances. Sociodemographic, pregnancy-related, and psychological factors have been related to depressive symptoms in the perinatal period. This study aims at (1) exploring personality and individual factors related with perinatal depressive symptoms, and (2) testing the mediating role of personality in the relation between characteristics of the woman’s family of origin and depressive symptoms.

**Methods:**

Women in the perinatal period admitted to the gynecology unit for motherhood-related routine assessments (n = 241) were included in the study. A survey on individual sociodemographic, clinical, and pregnancy-related factors was administered, also including the Edinburgh Postnatal Depression Scale (EPDS) and the BIG-5 personality test.

**Results:**

Couple conflict and neuroticism were independent and directly correlated with EPDS total score (respectively: B = 2.337; *p* = .017; B = 0.303; *p* < .001). Neuroticism was a significant mediator of the relation between the presence of a psychiatric disorder diagnosis in participant’s parents and the EPDS total score (indirect b = 0.969; BCCI95%=0.366—1.607).

**Conclusions:**

Couple relation and neuroticism traits are individual factors related to depressive symptoms in the perinatal period. The family of origin also plays an indirect role on perinatal depressive symptoms. Screening of these factors could lead to early recognition and more tailored treatments, ultimately leading to better outcome for the entire family.

## Introduction

Pregnancy is a major event involving neuroendocrine, anatomical, psychological and relational changes in the life of women. This complex transition can be characterized by vulnerability for mental disorders. Many clinical conditions such as anxiety, mood and psychotic disorders can affect women in the perinatal period, and perinatal depression (PND) shows a high prevalence [1, 2]: across nations and cultures PND has raised significant concerns due to prevalence rates ranging from 5 to 25% of women in the perinatal period [3–6].

Differently from the common, mild, and self-limiting “baby/maternity blues”, PND is an impairing depressive episode occurring during pregnancy or up to 12 months after childbirth [7, 8]. PND is especially alarming due to its multiple consequences affecting the mother as well as the couple and the mother-child relationship, with potentially severe consequences for the child’s development. Extensive studies have shown a causal relationship of symptoms of PND with child’s emotional development disorders and lower emotion recognition [9, 10], neuropsychological and cognitive deficits [11–13], internalizing and externalizing disorders [9, 14], sleep disturbances [9, 15], persistent lower growth [16] and worse long-term mental health outcomes [17, 18]. Ultimately, severe untreated PND can lead to self-harm, harming of the infant and, in tragic cases, to suicide and infanticide [19–21].

Existing evidence highlighted several factors which are related to PND. As well known by researchers and clinicians, the major neuroendocrine modifications happening during and after pregnancy play a role in the onset of PND [22, 23].

In addition to such biological factors, a growing body of evidence highlights the role of psychological aspects in PND. Patients’ remote and recent anamnesis have been related to PND in well-designed multivariate models. Specifically, a history of childhood trauma, poor marital quality, singlehood, domestic abuse, younger age, poor social support, and low socioeconomic status have been observed to be linked to PND symptoms [24–28]. Such evidence is consistent with epidemiological data on adverse childhood experiences, showing that certain early experiences (including exposure to psychological, physical, and sexual abuse as well as household dysfunction such as domestic violence, substance use, mental diseases, incarceration) are cumulatively related to greater degrees of adult illness burden [29].

Interestingly, patients’ personality has also been related to the onset of PND [30]. As frequently observed by specialists in the clinical practice, certain personality traits such as dependence, obsession, neuroticism, and severe self-criticism, are related to PND symptomatology [26, 31–34]. Emerging evidence also focused on the partner’s role and on how several aspects of the future father are closely intertwined to maternal mental health. The evidence shows a meaningful impact of fathers’ anxiety and depression, romantic and father-infant attachment style, material support, and co-parenting skills on the risk and evolution of maternal PND [35].

Only little research tested mediating and moderating etiopathogenetic models leading to PND, and no studies up to date (to the best of our knowledge) examined the possible mediating role of personality traits in the relation between patients’ family of origin history and PND symptoms. More specifically, early experiences and personality features are thought to be interrelated [36, 37], and it is possible that the experience of motherhood may interact with the women’s own experience of being a child taken care of [38]. Existing research accounting for the role of the family of origin in the prediction of PND suggests that having poor family relationships during childhood, as well as in their married life, and insufficient support from their families during pregnancy, increases odds of PND in multivariate models [39].

Considering the above, it is possible that certain characteristics of the family of origin can influence women’s personality, and that the interaction between such early experience and the occurrence of maladaptive forms of personality can ultimately lead to more severe depressive symptoms in the perinatal period. In order to elucidate the relationship between personality and depression in the perinatal period and in order to test the mentioned hypotheses, the first aim of the present study on women in their perinatal period was to explore personality traits and individual factors independently associated with depressive symptoms. The second aim of the present study was to test the mediating role of dysfunctional personality features in the relation between dysfunctional characteristics of the family of origin and severity of depressive symptoms; the findings of the first objective led us to consider the constructs of neuroticism and of having a parent affected by a psychiatric disorder within the analyses related to the second objective.

## Methods

### Subjects

The current study represents part of a larger clinical collaboration between the Perinatal Psychiatry out-patients Service and the Child-Neuropsychiatry Unit of “Umberto I” hospital of Rome. The project focuses on prevention and treatment of maternal mental disorders [40–42]. Recruitment started in February 2019 and ended in February 2020. It took place in the gynecology department of Policlinico Umberto I University Hospital, which is the largest public hospital of the metropolitan city of Rome. Recruitment was mainly performed by two psychiatrists from the Perinatal Psychiatry Service of the same Hospital: once a week, every woman consecutively admitted to the gynecology department for routine visits in the perinatal period were presented the project and proposed to take part in the study. Also, a minority of cases were proposed to participate in the study if the personnel of the gynecology unit asked for a psychiatric evaluation of specific cases. Inclusion criteria were: (1) being pregnant or within six months of giving birth; (2) accepting to take part in the research and give informed consent. Exclusion criteria were: (1) Diagnosis of moderate/severe cognitive impairment (2) insufficient Italian language skills (3) presence of severe acute psychiatric conditions requiring hospitalization (including suicidality). More in detail, eligible women were indicated by the gynaecologist and approached by the psychiatrist, following the gynaecological assessment, who explained the study aims and procedures and gathered the informed consent in case of acceptance of participation. Participants were administered the assessment tools as one bundle, to fill in privately as part of the gynaecological visit in one of the ward’s rooms. In case of doubts on specific items or other questions, participants could refer to the psychiatrist. Sociodemographic variables and pregnancy related variables were gathered from the gynaecological clinical chart as well as from a specifically designed sociodemographic data collection sheet.

A statistical power analysis was performed to determine the sample size using G*Power 3.1 software [43] based on the study objectives. The first objective of the study was to explore personality traits and individual factors independently associated with depressive symptoms; considering a potential amount of 24 predictors (as described below), the calculation indicated that, given a probability level of 0.05, a sample size of 169 was needed to provide a satisfactory statistical power (1–β = 0.80) to identify a medium effect size (f²=0.15) in a linear multiple regression model. The second objective of the study was to test the mediating role of neuroticism personality features in the relation between characteristics of the woman’s family of origin and severity of depressive symptoms; according to the sample size guidelines of Fritz and MacKinnon [44], in the mediation model, assuming medium effect sizes for two distinct pathways of the model (i.e., the effect of the independent variable on the mediator, and the effect of the mediator on the dependent variable), the analyses required a minimum sample size of 78 with the bootstrapping procedure to provide a statistical power of 0.80. Therefore, the current sample size (n = 241) has been chosen as it was considered to provide satisfactory statistical power in relation to the study objectives.

All participants gave their written informed consent to participate. The study received the approval of the Local Ethical Committee. At the end of the assessment, patients showing clinical signs of significant mental distress/mental disorders were proposed further clinical assessment in the out-patients service for perinatal psychiatry.

### Measures

The following information were collected:

- Sociodemographic variables: *age*, *education* (elementary-, middle-, high-school, and higher education), *relational status* (together, separated/alone), *professional status* (employed/unemployed);
- clinical variables (yes/no): anamnesis of *chronic medical illnesses*, anamnesis of *mental disorders*, ongoing *psychotherapy*, use of psychiatric *pharmacological therapy*, recent significant *griefs*, *psychiatric disorders in the family of origin* (parents only), history of *family conflict*, *partner’s mental disorders*, and *couple conflict*;
- pregnancy related variables: number *previous pregnancies*, previous *voluntary interruption of pregnancy* (VIP) (yes/no) or *miscarriages* (yes/no), use of *alcohol* (yes/no) or *tobacco* (yes/no) during pregnancy, *pregnancy complications* (yes/no).

The *Edinburgh Postnatal Depression Scale* (EPDS) was used to assess the presence and severity of PND symptoms. The EPDS is a cross-culturally validated 10-question form that a woman can complete in 2 to 3 min [45]. Total score can vary between 0 and 30. A score above 10 indicates “possible depression”, while patients scoring 12 or more are considered as suffering from a depressive illness of varying severity. The last item of the test enquires about suicidality and is used to identify patients with any degree of this symptom (i.e., scoring > 0). Cronbach’s α of EPDS for this sample was 0.835, indicating a good reliability [45, 46].

The *Big Five Inventory* (BFI) was used to assess main personality traits. It is a self-administered questionnaire, consisting of 44 items measuring the main characterizing traits according to the “Five Factor Model of Personality” [47]. They include: extraversion (8 items, which allow to identify traits such as sociability, assertiveness and positive emotionality) (Cronbach’s α = 0.756), agreeableness (9 items, referring to characteristics such as altruism, trust and tendency to provide support) (Cronbach’s α = 0.655), conscientiousness (9 items, referring to the ability to control impulses and self-discipline) (Cronbach’s α = 0.771), neuroticism (8 items, referring to traits of anxiety and negative emotionality) (Cronbach’s α = 0.755), and openness (10 items, identifying characteristics such as openness to experience, intellectual curiosity, aesthetic sense) (Cronbach’s α = 0.780). Each item consists of short sentences, patients were asked to assign a score on a scale from 1 = not at all agree to 5 = completely agree. Also, due to the different number of questions for each trait, each one has a different maximum score [48].

### Statistical analysis

Descriptive statistics are reported as numbers and proportions for categorical variables, and as means and standard deviations for continuous variables. Prior to analysis, data were screened for missingness. For the 39 cases showing some missing values (0.263% of the dataset, with big-5 and EPDS being 100% complete), automatic multiple imputation analysis was used to impute missing data.

An exploratory bivariate Spearman correlation analysis was performed to select, among the 24 individual and clinical collected variables, those variables significantly (*p* < .05) related to EPDS. Such variables were then used to construct a multivariate linear regression model to investigate factors independently associated with EPDS total score (dependent variable, objective 1). Data used in the regression respected the basic assumptions associated with a linear regression model that were tested (i.e., homoscedasticity, normal distribution of errors, multicollinearity).

Finally, based on the above-mentioned hypothesis and on the results of the correlation and regression analyses, we evaluated (using the original, non-imputed dataset) whether neuroticism (which is a dysfunctional personality feature found to be independently associated with EPDS) can operate as mediator in the relationship between history of a parent psychiatric disorder in the family of origin (which is a dysfunctional characteristic of the family of origin found to be bivariately, but not independently, associated with EPDS) and depressive symptoms (objective 2). Such analysis was performed using the extension “process” (model number four) of the Statistical Package for Social Science, with 10’000 bias-corrected bootstrapping procedure [49]. In the model, EPDS total score was the dependent variable, neuroticism was the mediator, and parent’s psychiatric disorder in the family of origin was the independent variable. Those variables which were independently associated with EPDS total score in the regression model (or which approached significance, *p* < .06) were included as covariates in the model.

## Results

The total sample consisted of n = 241 women in their perinatal period. Table 1 shows descriptive statistics of the sample. Mean age was 33.88 years (SD = 5.41; min = 16; max = 54). Only four (1.7%) patients were fully single mothers. Roughly half of the sample had a higher education, and the vast majority (82.2%) was employed. Although almost one out of five had a history of mental disorders, only few were under treatment with either psychotherapy or pharmacological therapy (7.9% and 6.2%, respectively). One in four patients reported a history of parent’s psychiatric disorder in the family of origin with one patient out of ten describing conflictual relations in the original family. Differently, 21 (8.7%) patients reported a partner’s psychiatric disorder, and the same percentage reported a conflictual relation with their partner.

**Table 1:** Descriptive characteristics of the sample

	*N*	%
Sociodemographic characteristics		
Status		
In a relation / Not in a relation	237 / 4	98.3 / 1.7
Education		
Middle school	22	9.1
High school	107	44.4
Higher education	112	46.5
Professional status		
Employed / Unemployed	198 / 43	82.2 / 17.8
Clinical characteristics		
Medical conditions (yes)	82	34
Alcohol during pregnancy (yes)	2	0.8
Smoking during pregnancy (yes)	10	4.1
Previous psychiatric disorders (yes)	44	18.3
Psychotherapy (yes)	19	7.9
Psychiatric medications (yes)	15	6.2
Recent griefs (yes)	61	25.3
Parent’s psychiatric disorder in the family of origin (yes)	60	24.9
Conflictuality in the family of origin (yes)	24	10
Partner’s psychiatric disorder (yes)	21	8.7
Conflict with the partner (yes)	21	8.7

Table 2 shows descriptive results related to the outcomes of interest. Overall, EPDS total score had a mean of 5.85 (SD = 4.63; min = 0; max = 23). A total of 31 patients (12.9) had an EPDS total score of 12 or more, suggestive of PND, and 12 (5%) reported suicidality as part of their condition. Personality traits are also presented in Table 2.

**Table 2:** Prevalence of perinatal depression, and personality traits

	*N*	*%*
**PND** (epds≥12)	31	12.9
	**mean**	**s.d.**
**EPDS total score**	5.85	4.63
**Big-five**		
Extraversion (max = 40)	28.07	5.25
Agreeableness (max = 45)	35.28	4.92
Conscientiousness (max = 45)	36.92	5.45
Neuroticism (max = 40)	21.34	5.59
Openness (max = 50)	38.05	6.09

**Note**

PND = Perinatal depression; EPDS = Edinburgh Postnatal Depression Scale; s.d.= Standard deviation

As already mentioned, bivariate associations were tested with Spearman correlation test. The following variables were significantly correlated to EPDS total score: previous psychiatric disorders (*ρ =* 0.165; *p* = .013); parent’s psychiatric disorder in the family of origin (*ρ =* 0.223; *p* = .001); conflict in the family of origin (*ρ =* 0.151; *p* = .020); conflictuality with the partner (*ρ =* 0.232; *p* < .001); partner’s psychiatric disorder (*ρ =* 0.137; *p* = .039); extraversion (*ρ=-*0.172; *p* = .007); agreeableness (*ρ=-*0.252; *p* < .001); conscientiousness (*ρ=-*0.243; *p* < .001); neuroticism (*ρ =* 0.463; *p* < .001). A table presenting all bivariate correlations is available upon request. Such variables significantly associated with EPDS total score were then included in the multivariate regression model showed in Table 3.

**Table 3:** Multivariate linear regression model predicting EPDS total score

		B	S.E.	t	*p*	95%C.I.	
						Lower bound	Upper bound
Previous psychiatric disorder		1.366	0.742	1.843	0.066	− 0.088	2.821
Parent’s psychiatric disorder in family of origin		1.134	0.706	1.607	0.110	− 0.262	2.530
Conflict in family of origin		1.073	0.920	1.166	0.244	− 0.730	2.876
Conflict with partner		**2.337**	0.982	2.381	**0.017**	0.413	4.262
Partner’s psychiatric disorder		0.820	0.418	1.959	0.050	0.000	1.640
Extraversion		− 0.078	0.054	-1.443	0.149	− 0.184	0.028
Agreeableness		− 0.009	0.060	− 0.156	0.876	− 0.127	0.108
Conscientiousness		− 0.022	0.053	− 0.424	0.671	− 0.126	0.081
Neuroticism		**0.303**	0.055	5.464	**< 0.001**	0.194	0.411

**Note**

S.E.= Standard error; C.I.= Confidence interval

In the regression model with EPDS total score as dependent variable (Table 3), having couple conflict, and neurotic personality traits were directly, independently correlated with EPDS total score (respectively: B = 2.337; *p* = .017; B = 0.303; *p* < .001), i.e., they were associated with EPDS also when controlling for the other variables (previous psychiatric disorder, a history of parent’s psychiatric disorder in the family of origin, conflict in the family of origin, partner psychiatric disorder, other analyzed personality traits). Having a partner with a psychiatric disorder approached significance (B = 0.82; *p* = .05).

**Fig. 1:**
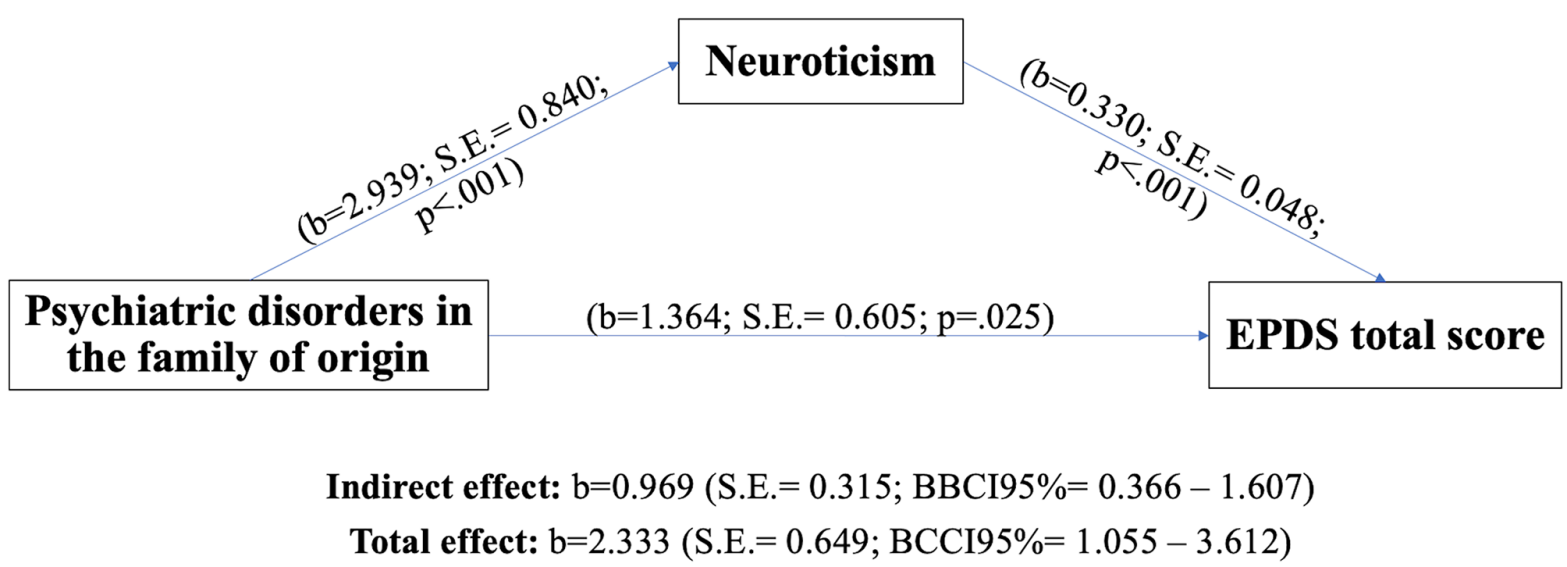
Mediation model of psychiatric disorders in the family of origin and EPDS total score through Neuroticism **Note** The model included conflict with the partner and partner’ psychiatric conditions as covariates. EPDS = Edinburgh post-natal depression scale. S.E.= Standard Error. BCCI = Bias-corrected confidence intervals.

In order to test our hypothesis that personality features mediate the relation between characteristics of the family of origin and depressive symptoms, we built a mediation model using EPDS total score as a dependent variable, neuroticism (i.e. a dysfunctional form of personality which was independently associated with EPDS in the regression model) as mediator, and parent’s psychiatric disorder in the family of origin (i.e. a dysfunctional characteristic of the family of origin which was significantly associated with EPDS by Spearman correlation but was not independently associated with EPDS in the regression model) as predictor. The model was controlled for the other variables independently significantly associated with EPDS total score (or which approached significance) in the regression model (i.e., conflictuality with the partner, partner’s psychiatric disorder). Figure 1 graphically shows the results of the mediation model. The total effect of parent’s psychiatric disorder in the family of origin on EPDS total score was significant (b = 2.3332; S.E.=0.6487; *p* = .0004; BCCI95%=1.0545—3.6119), and neuroticism partially mediated the relation between parent’s psychiatric disorder in the family of origin and EPDS total score with an indirect effect of b = 0.9688 (S.E.= 0.3154; BCCI95%=0.3655—1.6074).

## Discussion

In this research we studied individual features, personality traits and depressive symptoms in a group of women in their perinatal period. Results showed that the personality trait neuroticism (measured by means of the BIG5) and having a conflictual relation with the partner were directly and independently associated with PND symptoms (measured through the EPDS). Further, our hypothesis that personality (i.e., neuroticism) plays a mediating role in the relation between issues related to the family of origin and PND symptoms was supported; indeed, in the tested mediation model the effect of the presence of a parent’s psychiatric disorder in the family of origin on EPDS total score was significantly mediated by the personality trait neuroticism, even when the effect of partner-related factors were controlled for.

In relation to the first objective of the study, the results of the regression model suggested that neuroticism is strongly related to PND symptoms. Consistently, in our pool that there was no case of a participant scoring above the EPDS cut-off score for depression showing relatively low score in BIG-5 neuroticism scale. Building on this, couple conflict was also significantly related to EPDS, over and above confounding factors including a partner’s psychiatric disorder, possibly suggesting that conflictuality rather than a partner’s psychiatric disorder per se can play a role in perinatal depression. These findings raise the hypothesis that perinatal depression is a *sui generis* form of depression in which the symptomatology is closely related to personality and couple functioning. It is possible that, since pregnancy implies a profound redefinition of identity, dysfunctional aspects of personality in this period can facilitate the emergence of emotional distress. This is especially true when the partner is unable to function as containment and support within the couple. When these dysfunctional aspects are elicitated, depressive symptoms can worsen as maternal anxieties do not find adequate support neither at intrapsychic level (due to maladaptive personality traits such as neuroticism), nor at relational level (due to a problematic relation with the partner).

Confirming the relevance of the family of origin for the structuring of personality and relational style and for PND-related symptoms, the mediation model which was implemented to address the second objective of the study suggested that having a parent affected by a psychiatric disorder can have a role in explaining higher degree of neuroticism and, indirectly, increased depressive symptoms. As already mentioned in the Introduction, early experiences and personality features are interrelated, and it is possible that the psychological personality features related to the experience of pregnancy/motherhood may interact with the women’s own experience of being a child taken care of in the potential development of depressive symptoms.

Of relevance, other factors involved in the complex picture of peri-natal psychopathology, such as neuroendocrinological modifications happening during each phase of the pregnancy and in the pos-natal period [42, 50], have not been investigated in the present study, and future research is needed to link available knowledge on psychological and biological factors to understand the etiopathogenesis of PND and help clinicians in its prevention and treatment.

The present results need to be interpreted in the light of some limitations. First, the lack of longitudinal data does not allow to draw conclusions on risk factors leading to depressive symptoms, but it only allows to elucidate correlates of this condition; it would be of value to conduct long-term research on motherhood starting from the formation of the couple onward. Second, all anamnestic information provided by participants (e.g. presence or not of mental illnesses in the family of origin, conflicts with the partner) have not been verified and have been assessed without specific quantitative tools; also, EPDS and BIG5 scales are self-report measures, and this can be a potential source of bias, such as social desirability and inaccuracy biases; subsequently, the data included in the current study more closely show subjective rather than objective evaluations of participants on their own individual and psychological features, and should thus be interpreted with caution. Third, although the sample size is sufficient for the aims of our study, as suggested by *a priory* analyses, a multi-center study on a larger group could offer better representativity of the general population. Fourth, the present study is on depressive symptoms among women in the perinatal period, and it is thus not specifically focused on PND (i.e., the sample also included subjects without PND). Lastly, no information was available on the patients who refused to participate in the study, therefore no analyses of possible group differences between participants and non-participants could be performed. The present study also has some strengths: there was not *a priori* selection of patients, bringing to a more representative pool of women turning to a gynecology clinic; the assessment tools (EPDS and BIG5 scales) are widely validated and showed a good Cronbach’s α in the current sample; the analyses were performed statistically controlling for certain confounding factors.

In conclusions, this study contributes to elucidate the complex relationships linking personal risk factors, personality, and depressive symptoms in women in the perinatal period. The study highlights the relevance of the couple relation and of neuroticism traits as factors related to depressive symptoms in women in the perinatal period, as well as the role of parent’s psychiatric disorders in the family of origin on neuroticism and indirectly on PND symptoms. The present evidence highlights some relational, psychological, and familiar features which could be object of early screening and recognition of cases at greater risk of PND [51, 52]. It is important to recognize such cases and to offer effective treatments, possibly involving the partner (such as couple therapy), in order to avoid negative outcomes on the entire family system with potential short- and long-term effects also on the development of the newborn.

## Data Availability

The complete dataset could not be published online due to the data privacy section of the informed consent approved by the Local University Ethical Committee. Still, aggregated data and materials may be available upon reasonable request from the corresponding author.
